# A Pragmatist-Artifactualist view of experimental model organisms: the case of epigenetic inheritance

**DOI:** 10.1007/s40656-026-00748-z

**Published:** 2026-07-22

**Authors:** Mariano Martín-Villuendas

**Affiliations:** Departamento de Lógica, Historia y Filosofía de la Ciencia, Universidad Nacional de Educación a Distancia (UNED), Paseo Senda del Rey 7, 28040 Madrid, Spain

**Keywords:** Scientific models, Representationalism, Heredity, Environment

## Abstract

Model organisms have long occupied a central place in the life sciences, driving major discoveries and stabilizing knowledge about biological mechanisms conserved across taxa. However, their dominance has often eclipsed experimental model organisms—specimens chosen for their capacity to illuminate local hypotheses, usually associated with organism-specific features. This paper advances a twofold argument. First, I contend that overreliance on model organisms constrains the generation of novel hypotheses and narrows the conceptual landscape of biological inquiry. Using epigenetic inheritance as a case study, I show how this focus has systematically marginalized forms of inheritance that depart from conventional assumptions (i.e., germline/material overlap and transgenerational stability). Here, I concentrate on external intergenerational epigenetic inheritance. Second, I argue that merely vindicating the use of experimental model organisms is insufficient without examining the philosophical assumptions that structure their epistemic function. Specifically, I demonstrate that the representationalist conception—models as surrogates for target systems—permeates the conceptualization of experimental model organisms through the idea of representational function, thereby reinforcing framed trajectory-dependent inquiry. This representationalist conceptualization undermines the capacity of experimental model organisms to support framing exploratory inquiry oriented to test conceptually disruptive ideas, such as external intergenerational epigenetic inheritance. To overcome this limitation, I develop a Pragmatist–Artifactualist framework that reconceptualizes the epistemic role of experimental model organisms and restores their potential to expand the boundaries of biological understanding.

## Introduction

Model organisms constitute a cornerstone of contemporary biological practice (Ankeny & Leonelli, 2020). They have paved the way for crucial discoveries and provided the foundation for coherent research programs that have significantly advanced our understanding of fundamental biological mechanisms. In recent years, however, their use has expanded exponentially, often at the expense of what have been termed “Krogh organisms or experimental model organisms” (Dietrich et al., 2020; Green et al., 2018). The fundamental difference between these two categories lies in their cognitive orientation: while model organisms are designed to investigate generalizable biological processes, experimental model organisms are employed to explore local hypotheses, usually associated with organism-specific features. The preference for model organisms has traditionally rested on their alleged representational scope—that is, on how broadly findings obtained from a given organism can be projected onto a wider phylogenetic range.

Despite their many virtues, reliance on model organisms may constrain the conceptual landscape of biological inquiry if not complemented by alternative modeling strategies. Certainly, model organisms have been extraordinarily successful in consolidating robust knowledge about widely conserved physiological processes. However, their epistemic and pragmatic dimensions tend to favor deepening established lines of research—i.e., framed inquiry—, limiting opportunities to conduct research aimed at redefining or expanding the boundaries of the biological sciences—i.e., framing inquiry (Henne, 2023). Epistemically, model organisms are designed to highlight processes that are highly conserved across taxa, which naturally directs attention away from organism-specific phenomena. Pragmatically, their use typically requires tight alignment across multiple parameters—epistemic, historical, political-economic, and social—which complicates the exploration of questions that cast doubt on any of them. I will argue that experimental model organisms can help address this limitation: they may generate and stabilize innovative courses of investigation, thereby counterbalancing the tendency toward framed inquiry characteristic of model organisms. To illustrate this point, I will present the case of epigenetic inheritance (Jablonka & Lamb, 2005). I will show that the prevailing reliance on model organisms has led to a biased focus on specific forms of epigenetic inheritance—i.e., those that align with the hypotheses established by the dominant molecular-centered epigenetic research agenda (e.g., germline/material overlap and transgenerational stability conditions). This emphasis has marginalized cognitively promising forms of inheritance that challenge these assumptions, such as external intergenerational epigenetic inheritance.

However, merely shifting attention toward experimental model organisms is not, by itself, sufficient to ensure more innovative research agendas within biological practice. The epistemic value of experimental model organisms is often grounded in their representational function (Love & Travisano, 2013). This assumption is deeply tied to a broadly representationalist conception of scientific modeling—one that conceives models as surrogates of their target systems (Sánchez-Dorado, 2024). As various authors have emphasized (e.g., Andersen et al., 2019; Woodger, 1929), philosophical assumptions constitute unavoidable biases in science; they shape not only the rational reconstruction of scientific practice but also its very execution. I will argue that representationalism permeates the conceptualization of experimental model organisms through the idea of “representational function”, carrying significant limitations for the expansion of scientific understanding: it reinvigorates a framed path-dependent inquiry that privileges hypotheses aimed at refining established knowledge at the expense of more exploratory or disruptive ones—framing inquiry. Hence, it reintroduces the very epistemic constraints associated with the dominant use of model organisms. Reformulating the philosophical framework that underpins the epistemic role ascribed to experimental model organisms is therefore essential to enabling the formulation, articulation, and experimental corroboration of bold hypotheses that open new avenues of thought. To this end, I advance a Pragmatist-Artifactualist account.

In a nutshell, this paper advances two claims. First, conceptual understanding in biological research requires a pluralistic use of both model organisms and experimental model organisms, whose complementary strengths offset one another’s epistemic limitations. Second, achieving this pluralism demands revising the philosophical assumptions that guide how experimental model organisms are understood. Their current grounding in representationalism constrains their exploratory potential and reinforces framed research trajectories, thus blocking the possibility of a fine-grained conceptual articulation and empirical corroboration of disruptive hypotheses. To overcome this problem, I offer a Pragmatist-Artifactualist reconceptualization.

The paper is structured as follows. In the first section, I examine the defining features of both model organisms and experimental model organisms, clarifying their differences. I demonstrate how the former have come to dominate contemporary biological practice and argue that this dominance constrains conceptual innovation. Reorienting attention toward experimental model organisms, I suggest, may be fruitful for exploring novel experimental and theoretical directions. In the second section, I illustrate these considerations through current research on epigenetic inheritance. I contend that the prevailing reliance on model organisms has hindered the expansion of the concept of epigenetic inheritance by excluding a variant that challenges two entrenched dogmas in contemporary molecular-centered epigenetic research—i.e., the germline/material overlap and transgenerational stability conditions. In the third section, I argue that the skepticism surrounding a particular non-standard form of epigenetic inheritance, i.e., external intergenerational epigenetic inheritance, does not stem from its theoretical implausibility, but rather from a lack of experimental evidence. Specifically, I show how a representational conception of models has shaped the conceptualization of experimental model organisms via the notion of representational function, precluding the recognition of such phenomena. In the last section, I advance an alternative philosophical framework—i.e., Pragmatism-Artifactualism—that reconceives the epistemic function of experimental model organisms. This approach, I argue, allows experimental model organisms to fulfill their distinctive epistemic promise: testing the validity of innovative hypotheses, such as external intergenerational epigenetic inheritance, and, more broadly, expanding the space of biologically intelligible possibilities.

## Model organisms vis-à-vis experimental model organisms

In recent years, model organisms have received increasing philosophical and scientific attention. As Dietrich et al. (2014) demonstrate, their use in experimental studies has risen exponentially. While in the 1960s and 1970s the proportion was approximately 50% model organisms—50% non-model organisms, from 1990 onwards this trend broke in favor of the former, accounting for 75% of the articles. In fact, it is common to find statements favoring research focused on model organisms in leading disciplines. For example, Sommer (2009) has argued that conceptual clarity in evo-devo requires selecting a limited number of standardized model organisms and developing sophisticated, tailored toolkits.

The term “model organism” immediately evokes a familiar set of specimens associated with major milestones in twentieth-century biology—e.g., Drosophila melanogaster, Caenorhabditis elegans, Saccharomyces cerevisiae, and Mus musculus, among others. These species are included in the U.S. National Institutes of Health (NIH) compilation of thirteen “non-human mammalian and non-mammalian models,” created to guide funding priorities and promote efficient, stable exchange of biological data (e.g., genetic and phenotypic) and resources (e.g., strains or screening protocols).

There is a plurality of ways to conceptualize model organisms. For the present purposes, it is instructive to draw on the analyses presented by Sandra D. Mitchell (1997) in defining biological laws. She suggested three approaches: paradigmatic, normative, and pragmatic. The first two are of interest here. The paradigmatic approach would consist in providing examples of laws in physics and using them as a standard on which regularities in biology should be assessed. The normative approach would define law and then examine whether the generalizations conform. Considering model organisms, the problem with the paradigmatic approach is that the list has expanded over the years to incorporate “emerging” organisms ranging from the bee, the bat, the tomato, the snail, or the wallaby, among others (Ankeny & Leonelli, 2011; Cold Spring Harbor Press, 2009, 2010). This heterogeneity motivates the need for a normative account capable of synthesizing the central features these organisms share. Although it is extraordinarily difficult to establish a univocal definition of a model organism, there are certain points of agreement in the current literature (Ankeny & Leonelli, 2020; Dietrich et al., 2020; Minelli & Baedke, 2014).

First, model organisms exhibit a dual ontology (Ankeny & Leonelli, 2020, p. 20). On the one hand, they constitute real biological entities whose existence derives from the natural mechanisms that drive evolution. On the other hand, they are artifactual entities deliberately modified to meet research demands. In other words, they are the product of complex standardization processes that may vary according to the research goal.

Second, model organisms possess a broad representational scope (Ankeny & Leonelli, 2011; Green et al., 2018). This concept refers to how extensively the results obtained with an organism can be projected to other organisms. Research conducted on model organisms allows us to bring to light certain basic physiological processes assumed to occur similarly in other organisms. Data, results, and theories obtained regarding ontogenetic, physiological, or evolutionary details can be extrapolated following the order of phylogenetic proximity: first, to other species of the concerning genus; second, to other genera; then to members of the corresponding phylum, and so on (Minelli & Baedke, 2014). Extrapolations will be more reliable the closer the phylogenetic proximity (Levy & Currie, 2014). This feature imbues model organisms with an incontestable epistemic value: they pave the way for advancing an integrative science that provides a global picture of core biological processes shared by different organisms (Jenner & Wills, 2007).

Third, they possess certain features that make them suitable for efficiently and stably pursuing relevant research questions. For example, they have small physical sizes, relatively simple genomes, uniform development, genetic stability, simple reproductive cycles, fast life cycles, high fecundity rates, or low costs of reproduction, maintenance, and transport. Inevitably, trade-offs arise: depending on pragmatic considerations, researchers may prioritize some features at the expense of others. Additional contextual factors—such as availability, ethical constraints, or commercial uses—also influence organism choice (Dietrich et al., 2020).

Finally, model organisms anchor entire research communities (Ankeny & Leonelli, 2011). The NIH list was designed precisely to consolidate stable, collaborative networks. Such stability requires the alignment of epistemic, historical, social, and political-economic elements (Ankeny & Leonelli, 2020, pp. 41–42). For this reason, Ankeny and Leonelli have associated model organisms to the idea of repertoire:

well-aligned assemblages of skills, behaviors, and material, social, and epistemic components that groups may use to practice certain kinds of science, and whose enactment affects the methods and results of research, including how groups practice and manage research and train newcomers. (Ankeny & Leonelli, 2016, p. 20)

Communities that employ model organisms hold a tacit agreement regarding theoretical and conceptual assumptions, share questions that should guide research, stable (cyber)infrastructure of information exchange (e.g., databases, newsletters, and/or journals), behaviors (exchange of data and techniques), strategies for obtaining funding and processes of standardization and centralization of the production, use and dissemination of specimens.^1^ The latter explains the existence of a pragmatic component involved in the preference for certain model organisms: researchers are incentivized to work with model organisms that fit the experience and history of scientific success to accomplish a given task (Burian, 1993). The resulting preference for model organisms thus has an epistemic dimension—model organisms possess the features needed to yield generalizable insights—and a pragmatic dimension—they align with the established expertise, infrastructures, and success records of scientific communities (Barresi & Gilbert, 2020; Bertile et al., 2023).

Bearing these considerations in mind, Ankeny and Leonelli (2018) have formulated an uncontroversial definition of model organisms:

non-human species that are easy to breed and maintain in large numbers under laboratory conditions and which are extensively studied in order to understand a range of biological phenomena, with the hope that data and theories generated through the use of the model will be applicable to other organisms. (Ankeny & Leonelli, 2018)

Drawing on the normative approach is central to differentiating model organisms from another class of organisms that play an equally central epistemic role: Krogh organisms or experimental model organisms^2^ (Bolker, 2009). The latter have been conceptualized as tractable systems that allow the study of particular biological questions in controlled research environments (Ankeny & Leonelli, 2011; Green et al., 2018). Their cognitive relevance does not derive from their capacity to provide general principles but from the fact that their particular features make it possible to address questions of interest by making them experimentally accessible. These organisms facilitate the construction of a suitable experimental environment, allowing researchers to perform the manipulations required to evaluate a given hypothesis.

Drawing on the epistemic and pragmatic dimensions already introduced, it is possible to clarify the differences separating model organisms from experimental model organisms. Epistemically, model organisms are considered valuable because of their representational scope; studying them enables inferences concerning physiological processes widely shared across taxa. A good example is the use of Drosophila melanogaster to describe general principles of developmental regulation extrapolable to other metazoans.^3^ Specifically, the identification of segmentation genes crucial for the formation of the body pattern of bilateral animals (Nüsslein-Volhard & Wieschaus, 1980; see also Bolker, 2014b). By contrast, experimental model organisms are epistemically salient not due to their capacity for extrapolation but because of their representational function. They are chosen precisely because they exhibit the features needed to make the question under investigation experimentally tractable. An example is the use of frogs, particularly Rana pipiens, by Krogh to conduct research on the mechanisms of gas exchange, osmotic regulation, and the physiology of capillary function (Larsen et al., 2021). He chose this organism because of an exceptional physiological property not necessarily pervasive in other vertebrates: its skin and epithelial tissues exhibit unusually high and experimentally accessible permeability. In this context, no prior attempt was made to fix the representative scope of the conclusions—an issue addressed empirically on a case-by-case basis.

Pragmatically, model organism-oriented research privileges features associated with standardization—e.g., small physical size, simple genomes, uniform development, ease of supply, durability, or low costs of reproduction, maintenance, and transport—over those required to mimic the fine-grained mechanisms of interest. Indeed, assuring the latter may even be counterproductive. As Bolker notes: “we should seek models that are not too highly specialized and that retain ancestral character states—or the modal state for extant members of a taxon—with respect to the subject of inquiry” (Bolker, 2009, p. 491). To yield generalizable insights about biological mechanisms that extend across taxa, model organism research must secure a rigorous process of standardization to ensure that data can be reliably compared, circulated, and integrated across research contexts (Ankeny & Leonelli, 2020). Such integration demands an alignment among epistemic,^4^ historical,^5^ political-economical,^6^ and social^7^ parameters. Without such alignment, the integrative aims of model organism science cannot be realized. By contrast, these considerations do not apply to experimental model organisms, as their representational function outweighs representational scope. Experimental model organisms require alignment only between the organism’s material properties, the research goal, and the relevant performative capacities of researchers. They do not require the robust, community-wide alignment characteristic of model organism research.

Two clarifications are in order. First, the term “experimental” is reserved for Krogh organisms because their distinctive properties (physiological, ecological, or evolutionary) permit targeted manipulations within a research environment to address particular research questions. They function as genuine “experimental artifacts”: their properties provide a gateway for experimentally evaluating hypotheses about specific processes not necessarily conserved across taxa. By contrast, the epistemic value of model organisms lies not in enabling targeted interventions but in enabling broad generalizations. Although model organisms are “experimental” in a broad sense, they are not experimental in the narrow sense just outlined.

Second, the boundary separating the two categories is context-dependent: the same organism may be classified one way or another depending on the phase or type of research. A specimen initially used as an experimental model organism may become a model organism once theoretical and performative knowledge, technical infrastructures, and communities of practice coalesce around it, enabling standardization and data exchange (Ankeny & Leonelli, 2020). Conversely, a model organism may serve as an experimental model organism when its specific material properties become salient for answering a local question. No organism is inherently or absolutely a model organism or an experimental model organism; categorizations depend on the contextual nexus in which the organism is situated.

The privileged status granted to model organisms has generated certain undesired consequences. Most notably, it displaces experimental model organisms from central roles in research (Dietrich et al., 2014) or promotes attempts to transform all experimental model organisms into standardized model organisms (Leonelli & Ankeny, 2013). Yet multiple authors highlight the epistemic importance of experimental model organisms for conducting research aimed at advancing our understanding of biological reality. First, their use may expose and compensate for the biases associated with using traditional model organisms. For example, due to the interest in fundamental internal biological processes, research in model organisms tends to standardize certain higher-order factors such as environment or epigenetics, providing a distorted picture of their importance and centrality in the ontogenesis (Bertile et al., 2023; Collins et al., 2007). Second, they bring biological diversity into view. Model organism-centered research focuses on exploring the mechanisms that bind all life forms together, overlooking the mechanisms that separate and confer them with their identity (Green et al., 2018; Leonelli & Ankeny, 2013).

Here, I develop a further reason to reorient research attention toward experimental model organisms. Collins et al. (2007) argue that model organisms function as “comfort zones,” discouraging engagement with difficult or conceptually disruptive questions. I show that overreliance on model organisms risks constraining conceptual innovation by privileging integrative, well-established aims over exploratory ones. Epistemically, research centered on model organisms tends to focus on processes that are highly conserved across taxa. This focus, while epistemically advantageous, naturally diverts attention from organism-specific or local phenomena, which may be critical for addressing boundary-expanding questions. Pragmatically, model organisms research requires a tight alignment across multiple parameters—epistemic, historical, social, and political-economic—which ensures robust and reproducible investigations of these broadly conserved processes. However, this alignment can marginalize alternative hypotheses that challenge elements of the established framework or underfund their respective research programs. While model organisms have undoubtedly contributed to the consolidation of robust knowledge, exclusive reliance on them is epistemically shortsighted. Addressing boundary-expanding questions and investigating potential exceptions is therefore essential for advancing our understanding of biological complexity. In what follows, I examine how experimental model organisms provide key epistemic tools for opening new avenues of inquiry, focusing on contemporary research on epigenetic inheritance (Jablonka & Lamb, 2010).

## Exploring new avenues of thought: external intergenerational epigenetic inheritance

In the early days, epigenetics research relied heavily on organisms inherited from the long-standing tradition of genetic experimentation to test emerging hypotheses (Allis et al., 2007). Although working with well-characterized organisms offered clear methodological advantages, it also tended to channel data collection and interpretation along lines shaped by classical genetic assumptions (Jablonka & Lamb, 2020, pp. 14–18). As researchers began to question some of these assumptions, it became increasingly important to examine organisms whose material, ecological, and phylogenetic characteristics were better suited to evaluating the plausibility of the new hypotheses being advanced. In practice, this required approaching certain specimens under what I describe as the logic of experimental model organisms. The epistemic and pragmatic dimensions of experimental model organisms proved crucial for challenging several deeply entrenched assumptions in genetic research. As noted above, epistemically they are oriented toward testing highly specific hypotheses rather than establishing the conservation of mechanisms across taxa—an orientation that was particularly important at this stage of inquiry. Pragmatically, their use did not require the extensive alignment among parameters that typically structures research centered on model organisms. As a result, they provided a flexible investigative strategy for exploring novel possibilities that could not easily be accommodated within established model organism frameworks. The following cases illustrate this transition.

A prevalent assumption held that genetic variation necessarily preceded epigenetic variation, with the latter causally dependent on the former (Richards, 2006). Studying experimental model organisms and analyzing their ontogeny under hitherto unconsidered ecological conditions demonstrated that epigenetic variation could precede genetic variation. Epigenetic variation was shown to be the main source of variation in asexually reproducing clonal populations exhibiting minimal genetic variation (Dybdahl & Kane, 2005). Similarly, epigenetic marks were identified as fundamental sources of variation enabling populations with low genetic diversity to colonize novel environments (Schrey et al., 2012).

Another common belief concerned the impossibility of transgenerational germline epigenetic inheritance (Heard & Martienssen, 2014). Decades of research in genetics, especially in the context of mammalian model organisms, reinforced a dominant research orientation centered on genetic inheritance, often relegating alternative mechanisms—such as the epigenetic—to the margins of mainstream inquiry (Baedke, 2018; Deichmann, 2016; Morange, 2002, 2020). This belief rested on two ideas: (1) epigenetic reprogramming—two rounds of epigenetic mark erasure, the first during gamete production and the second in the embryo—; (2) the early separation that takes place between the somatic line and the germline (Futuyma, 2017, p. 94). Research with specific experimental model organisms stressed the limitations of both ideas. Regarding the first, studies demonstrated that the paternal methylome is largely preserved during early embryogenesis in certain organisms—e.g., Danio rerio (Jiang et al., 2013)—or only partially reprogrammed—e.g., invertebrate deuterostomes and tunicates (Vogt, 2022). Regarding the second, it was discovered that environmentally induced epigenetic marks can be easily transmitted to the next generation in plants. Recently, a communication system (vesicles containing modified RNAs) has been found in certain mammals that can transmit epigenetic information from soma cells to the germline (Sciamanna et al., 2019).

However, this initial interest in experimental model organisms gradually waned. As epigenetics became more firmly established as a field, researchers increasingly sought to demonstrate that the processes identified were not confined to particular cases but were shared across a broad range of taxa (Boskovic & Rando, 2018; Fitz-James & Cavalli, 2022). This shift prompted a transition toward model organisms—or to the reinterpretation of experimental model organisms within the logic of model organism research. One plausible explanation for this shift is the desire to consolidate the discipline by concentrating research on organisms that could help stabilize its conceptual framework and socio-institutional infrastructure (Clarke et al., 1992; Creager et al., 2007).

Turning to inheritance, epigeneticists currently agree on two incontrovertible conditions as defining evolutionarily relevant epigenetic inheritance:

1. Transgenerational stability condition. To consider an element as “heritable”, it must significantly impact evolutionary dynamics. This, in turn, implies that it must persist over generations (F0-F2/F0-F3).
2. Germline/material overlap condition. Given the aforementioned condition, only elements contained in the germline that contribute to the reconstruction of an adaptive trait are considered heritable.

Commitment to these conditions has reinforced the view that evolutionarily significant epigenetic inheritance is confined to mechanisms satisfying both transgenerational stability and germline/material overlap (e.g., Daxinger & Whitelaw, 2012, p. 154; Fitz-James & Cavalli, 2022, p. 325). Consequently, much contemporary research has focused on employing organisms from the perspective of model organisms: well-characterized specimens studied under established and controlled experimental conditions, which allow researchers to generate generalizable principles about the mechanism. This approach aims not only to characterize the specific features of the underlying processes—such as methylation, histone modifications, and RNAs—but also to determine the contexts in which they arise and operate, and to assess their prevalence across different cases. For instance, several studies in C. elegans demonstrate that environmental factors can induce epigenetic changes that persist and modulate phenotypes across generations (Rechavi et al., 2014). Work with planaria (Neuhof et al., 2016) and M. musculus (van Steenwyk et al., 2018) further illustrates that environmental exposures can produce epigenetically mediated transgenerational phenotypic effects.

Undoubtedly, these studies have been fundamental in challenging certain assumptions rooted in the genetic inheritance tradition, such as the Weismann barrier and the strict separation between inheritance and development. They have also been crucial in articulating a robust research program focused on detailing the processes underlying meiotic transgenerational epigenetic inheritance common to a wide variety of organisms, assessing its prevalence. However, this has come at the cost of reinforcing a framed inquiry that considers transgenerational stability and the germline/material overlap as fundamental tenets. The crux of the matter is that focusing all research efforts exclusively on this approach risks limiting our understanding of the nature of inheritance by overlooking possible forms of epigenetic transmission that may be evolutionarily important, despite deviating from these two conditions.

In recent years, several authors have questioned the universality of transgenerational meiotic epigenetic inheritance. First, they have challenged the molecularist approach—i.e., methylation, histone modification, or RNAi—, arguing that attention should also be given to the developmental processes underlying trait (re)construction: physiological feedbacks, cellular interactions, tissue constraints, and environmental inputs (Schwab et al., 2017; Sultan, 2015). Second, it has been argued that germline-transmitted elements are not the sole contributors to trait reconstruction. Understanding phenotypic trait (re)construction requires considering a heterogeneous developmental matrix that may extend beyond the lineage itself (e.g., Newman & Müller, 2000; Oyama et al., 2001; Veigl et al., 2022). Third, the idea that only long-term inheritance can influence evolutionary dynamics has been questioned (Lala et al., 2024; Stotz & Griffiths, 2016). Since selection acts on phenotypic differences, epigenetically mediated trait reconstruction will be evolutionarily relevant as long as it concerns traits considered central to survival/reproduction and the environmental trigger is intergenerationally stable. Two scenarios illustrate this point. First, it may offer more efficient evolutionary strategies than genetic transmission in fluctuating environments, reducing the risk of lineage extinction (Hu & Barrett, 2017; Lala et al., 2024). Second, and related to the previous point, it could facilitate the colonization of new ecological niches, promoting the divergence of populations (Flatscher et al., 2012). This is particularly relevant because environmentally induced epigenetic variation can appear simultaneously in multiple individuals, making adaptive epigenetic variants less vulnerable than mutations to loss through genetic drift or population extinction (Lala et al., 2024).

**Fig. 1:**
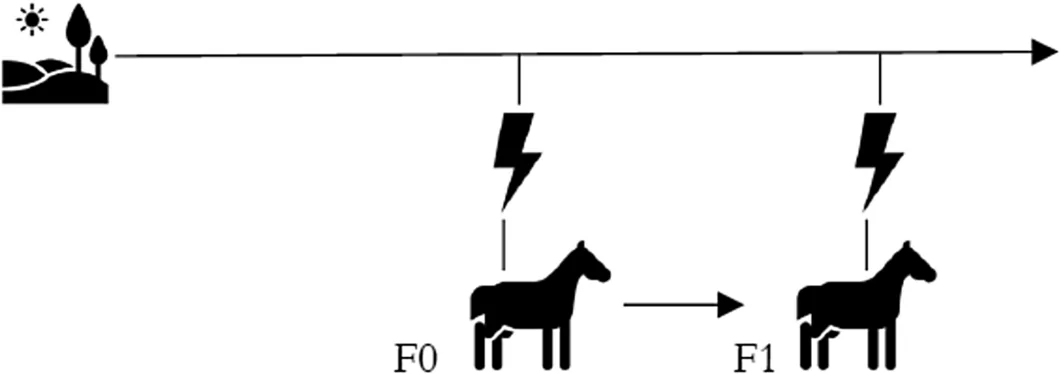
The (re)appearance of the adaptive trait in the lineage results from the intergenerational action of environmental factors that have a specificity in triggering key epigenetic processes involved in the ontogenetic reconstruction of the trait. It is not necessary that the environmental factor is the same, only that it has the same specificity with the epigenetic processes that produce the adaptive trait

These criticisms have gained momentum with the resurgence of a largely overlooked tradition within epigenetic thought^8^: organicism (for a conceptualization of the molecular and organicist epigenetic traditions, see Deichmann, 2016; Goldberg et al., 2007; Haig, 2004; Lederberg, 2001; Peterson, 2016). *Grosso modo*, organicists argued that development is an epigenetic process structured through dynamic interactions among components at multiple regulatory levels (Nicholson & Gawne, 2015; Peterson, 2016). Central to this view are not only interactions among internal factors but also the integration of materials and energy from the environment through a series of transformations (Ritter, 1919, p. 313; Woodger, 1929, p. 332). Understanding the reconstruction of phenotypes across generations requires considering the various factors shaping ontogenetic trajectories and the interactions between the various regulatory scales that shape and maintain the integration of organismal form. Any substance influencing development—e.g., cytoplasm or environmental components—can be regarded as a physical basis of heredity (Child, 1925, p. 132; Ritter, 1919, p. 64) (Fig. 1).

Building on this organismal perspective, the possibility emerges to consider a mechanism of epigenetic inheritance that leaves aside molecularism and the two widely accepted conditions mentioned above, i.e., germline/material overlap and transgenerational stability conditions:

*Def. External intergenerational epigenetic inheritance*. It encompasses all developmental processes—molecular and non-molecular—that trigger the intergenerational (F0-F1) reconstruction of certain adaptive traits within a lineage in response to highly specific environmental signals. Therefore, the mechanism of inheritance transcends the contours of the organism by including temporary but specific environmental signals as necessary determinants of key ontogenetic processes involved in the reconstruction of particular adaptive traits.

The validity of this notion of inheritance requires empirical support. I suggest that, to assess the viability and prevalence of this mechanism, research should make a reverse transition—from model organisms back to experimental model organisms. This shift is necessary because the mechanism in question challenges the central assumptions of canonical epigenetic research and is likely not widespread across taxa, but rather represents a localized phenomenon that must be assessed on a case-by-case basis. Consequently, selecting organisms that exhibit the appropriate material, phylogenetic, and ecological features to test the core intuitions of the hypothesis is essential. Namely, that certain traits are reconstructed externally through temporally specific interactions between internal mechanisms and environmental inputs, and that their intergenerational persistence can influence evolutionary dynamics. Experimental model organism research is particularly well-suited for this task, given its epistemic and pragmatic dimensions. Although this movement is not yet widespread, initiatives are already emerging. Consider the following examples.

Thorson et al. (2017) showed that environmentally induced epigenetic differences can generate adaptive variation and facilitate ecological niche colonization in genetically impoverished, asexual populations of the freshwater snail Potamopyrgus antipodarum. Morphological divergence and epigenetic variation were observed among populations in distinct habitats, with shell shape differences consistent with adaptation to water current velocity. Genome-wide DNA methylation differences were linked to the development of these adaptive traits. Similarly, Stajic et al. (2019) investigated genetically uniform yeast populations adapting to a toxic chemical, demonstrating that multigenerational heritable silencing via epigenetic inheritance promoted survival and growth, thereby enhancing the availability of adaptive mutations. In a related study, Stajic et al. (2022) tested the adaptive value of low-stability mechanisms under laboratory conditions. They found that under fluctuating environments, clones with epigenetically controlled URA3 expression were favored, whereas genetic changes predominated in stable environments. These findings highlight that greater stability is not universally a superior evolutionary strategy.

In short, a tradition of biological thought—i.e., organicism—, a core of contemporary theoretical studies—e.g., DST, evo-devo, or eco-devo—and some experimental results demonstrate the plausibility of external intergenerational epigenetic inheritance. I claimed that central to legitimizing this hypothetical mechanism of epigenetic inheritance is the deliberate use of experimental model organisms possessing the characteristics required to test it. Only through them can the mechanisms (processes regulating trait encoding) and prevalence (which organisms and ecological contexts) be elucidated. However, vindicating the transition towards experimental model organisms is not, in itself, sufficient: it must be coupled with a philosophical analysis clarifying how these models generate epistemically valuable insights. In the next section, I will show how ignoring this point risks undermining the very revolutionary potential that experimental model organisms provide.

## The representational view of experimental model organisms

In Sect. 1., I argued that research focused on model organisms and experimental model organisms differ epistemically in virtue of privileging representational scope and representational function, respectively. Model organisms contribute to understanding by enabling inferences that extend across a wide range of taxa. By contrast, experimental model organisms—the *representans*—are selected because they instantiate the necessary characteristics to shed light on the *representatum*—e.g., the epigenetic inheritance mechanism discussed above. Consequently, their epistemic value rests on their representative function: the capacity of the model system to stand for and illuminate relevant aspects of the biological phenomenon under study. The “representational function” attributed to experimental model organisms echoes the broader representationalist framework that currently dominates philosophical accounts of scientific modeling.

This continuity is not accidental. Scientific practice is never conducted in a vacuum, but it is shaped by what may be termed philosophical biases—commitments ranging from representationalism, realism, to veritism, among others. As Andersen et al. (2019) argue, such philosophical biases are not optional add-ons but constitutive elements of scientific reasoning. They scaffold research practices by guiding the formulation of questions, the establishment of evidential standards, and the determination of what counts as a satisfactory explanation of a phenomenon. While not all forms of scientific research necessarily adopt a representational lens—as Levy and Currie (2014) note regarding model organisms—research centered on experimental model organisms clearly does. These organisms are selected and manipulated precisely because they are taken to stand in for key features of interest in the *representatum*. To appreciate to what extent the representational assumption pervades the conceptualization of the epistemic role of experimental model organisms, it is instructive to briefly introduce the fundamentals of the representationalist view within the debate on scientific models.

Currently, it is widely accepted that models sensu lato are irreplaceable tools for conducting scientific research. Over the last few decades, philosophers have sought to clarify why models play this central role. As said, a growing consensus holds that their epistemic value resides in their representational capacity (e.g., Gelfert, 2016). In fact, this is the reason why the debate about the epistemic function of models has been prone to collapse into the debate around scientific representation (Frigg & Nguyen, 2020). Under the representationalist umbrella, model systems have been conceived as interpreted, idealized, and tractable devices that researchers intentionally construct to represent certain key aspects of a given target system in the natural world. In other words, they constitute intentionally modified versions of phenomena that reduce their complexity, allowing access to certain features deemed relevant. Elsewhere (Martín-Villuendas, 2024), I have distinguished two major variants within representationalism.^9^

The first grounds representation in shared features—typically analyzing the relationship between the *representans* and the *representatum* in terms of similarity or morphism (e.g., Giere, 2006; van Fraassen, 1980). Here, one can find informational views, which reduce representation to an objective parameter that relates properties of vehicles to those of the target, and pragmatic views, where it is claimed that illuminating the representational relationship requires bringing out the intentional judgments of users (Chakravartty, 2010). Currently, the emphasis placed on pragmatic and contextual aspects is the dominant one in the general philosophy of science and the very life sciences (e.g., Bolker, 2014a). In fact, a basic and intuitive form of this version seems to be the one operating in the case of experimental model organisms.

The second variant of representationalism emerged in response to the challenge posed by “holistically distorted models”—models that systematically distort all elements of the target, including the ones that make the difference, to introduce mathematical tools to yield epistemically valuable insights. Proponents of this view argue that representationalism should not be tied to literal accuracy (e.g., Rice, 2021). Models can represent by uncovering stable patterns of behavior, which are grounded in robust inferential relations of counterfactual (in)dependence. Thus, it is possible to guarantee inaccuracy while keeping intact the representational commitment. This second variant of representationalism does not usually operate in the case of experimental model organisms, being more prevalent for alternative models such as computational ones.

The representational attitude is not merely a rational reconstruction of scientific practice or a philosophical prescription. In certain cases, such as experimental model organisms, it seems to be a constitutive element that renders the use of certain scientific model epistemically intelligible (Bolker, 2009). Recognizing the influence of philosophical biases such as representationalism should not lead us to try to eliminate or minimize them. As I have said, these commitments are central to productively organizing scientific practice, directing attention toward specific questions, explanatory strategies, and evidential standards. The critical issue lies in evaluating whether the operative philosophical principles are the most appropriate for guiding particular research practices. Almost a century ago, Woodger (1929) demonstrated that many of the enduring debates in biology—such as Mechanism versus Vitalism, Organism versus Environment, and Structure versus Function—were rooted in certain philosophical compromises. Extending this insight to contemporary research, my central claim is that the representational view of experimental model organisms constrains their epistemic potential and intrinsic virtue. Specifically, it encourages a framed path-dependent inquiry in which experimental model organisms are primarily used to articulate fine-grained details of accepted views rather than to explore, test, or refine unconventional hypotheses. In what follows, I will examine this argument in detail.

Representational approaches demand that the explanandum be sufficiently well specified to conduct research. Precisely defining the explanatory target is crucial, as it frames both the inquiry and the subsequent search for explanations. As Ross observes: “A scientific explanation cannot be provided until the explanatory target is sufficiently clear” (Ross, 2024, p. 6). In practice, this entails having substantive prior knowledge of the target system, including the plausibility of the hypotheses, where and when it occurs, and the mechanisms that account for it. Consider research on epigenetic inheritance. A representational framework presupposes clarity regarding: (i) the entities and processes potentially involved in the (re)construction of adaptive traits; (ii) the environmental cues triggering the (re)construction of the adaptive trait; (iii) the specificity connecting epigenetic processes, environmental cues, and phenotypic outcomes; and (iv) the systems in which these processes occur. Such conditions are typically met in research programs aimed at elaborating or refining existing knowledge. A clear example is the research carried out on germline transgenerational epigenetic inheritance (see, e.g., Fitz-James & Cavalli, 2022; Jablonka & Lamb, 2010). By contrast, in many cases—particularly in research aimed at expanding the contours of the discipline—this prior knowledge is simply not available.

The representationalist view aligns with what can be termed “framed inquiry,” a distinction rooted in the pragmatist tradition and brought into focus in contemporary philosophy of science by Henne (2023). Framed inquiry refers to investigations conducted within a well-defined conceptual framework that delineates both how to ask and how to answer questions. However, scientific research does not collapse into framed inquiry. A second type of research is present, characterized not by elaborating established frameworks, but by revising or expanding them: framing inquiry. Hypotheses in framing inquiry are often vague, tentative, or even highly improbable. Mechanistic understanding of the processes underlying the phenomenon is incomplete, relevant ecological conditions may be unknown, and the range of organisms in which it occurs may be uncertain. Furthermore, it is also possible that we do not have a deep mechanistic understanding of the putative target to which it applies. Importantly, such an inquiry is not epistemically defective. It should be seen as a complementary and equally necessary counterpart to framed inquiry: while framed inquiry provides stability by consolidating knowledge within well-defined conceptual frameworks, framing inquiry introduces dynamism by opening new avenues for conceptual and empirical exploration. In my view, framing inquiry characterizes the epistemic role of experimental model organisms. Their value does not lie in representing well-established targets but in making poorly understood phenomena experimentally accessible. In other words, the goal is not to articulate the details of the framework, but to extend or reformulate its contours.

Interpreting experimental model organisms through a representationalist lens risks collapsing framing inquiry into framed inquiry, thereby undermining the exploratory potential of experimental model organisms to expand the existing conceptual framework of heredity. Representationalism reinvigorates framed inquiry, leading to path dependence research: hypotheses aimed at elaborating details of already accepted knowledge are prioritized, well-characterized ecological contexts become the default research settings, and target systems with clearly delineated contours are preferentially studied. Phenomena that fall outside the standard framework—e.g., the mechanism of external intergenerational epigenetic inheritance—are treated as anomalies rather than as opportunities for conceptual expansion. As a result, such investigations are abandoned, along with the possibility of obtaining empirical evidence that would eventually allow us to determine the plausibility of this mechanism, specify its properties, and determine its scope. It is important to note that a lack of epistemic precision—i.e., we do not have a clear specification of the hypothesis and a clear understanding of the target to which it applies—does not imply a lack of epistemic value. Similarly, a limited general instantiation—i.e., it occurs in restricted organisms and situations—does not entail that it is negligible or epistemically uninformative. Mechanisms such as external intergenerational epigenetic inheritance, even if taxonomically restricted or ecologically contingent, may illuminate aspects of heredity and development inaccessible within existing frameworks, thereby refining our understanding of evolutionary scenarios and physiological processes.

In summary, vindicating the use of experimental model organisms without critical reflection on the philosophical assumptions guiding their use can incur epistemic costs. Representationalism, though valuable for certain types of research, may hinder the value of practical inquiries oriented to test disruptive hypotheses that still have vague contours, thus consigning them to ostracism. I have argued that this is particularly evident in the case of experimental model organisms, which are often conceptualized in terms of their representational function. This limitation, I have suggested, stems from the representationalist tendency to collapse framing inquiry into framed inquiry. If biological research is to remain responsive to conceptual novelty—and here the mechanism of external intergenerational epigenetic inheritance is an example—it is essential to preserve the distinction between framed and framing inquiry, reconsidering the role assigned to experimental model organisms in structuring scientific understanding. This is the task I undertake in the final section.

## A Pragmatist-Artifactualist view of experimental model organisms

Representationalism is not the only framework for shedding light on the epistemic role of models. Several alternative accounts offer conceptual resources that speak directly to the criticisms outlined above. Sanches de Oliveira, for example, advances a form of radical artifactualism that grounds the epistemic value of models in their capacity to scaffold problem-solving practices and support situated sense-making (Sanches de Oliveira, 2022, p. 30). Similarly, Gelfert (2024) has advocated a view that challenges targetism—the belief that models should be thought of as the kind of thing defined by something else they refer to. Drawing on enactive and phenomenological traditions, he instead conceptualizes models as mediating devices that structure particular modes of embodied engagement with the world.

While a critical assessment of these frameworks that identifies commonalities and differences is a valuable topic on its own,^10^ the remainder of the manuscript will be devoted to developing an original account grounded on two complementary lines of thought: Dewey’s pragmatist account of experience and Knuuttila’s artifactualist conception of models. In what follows, I will evidence how this Pragmatist-Artifactualist account undermines two representationalist-connected entrenched assumptions that constrain the development of framing inquiries oriented at expanding the contours of disciplines.

First, this approach rejects the idea that the epistemic value of models lies in their representational function. According to this Pragmatist-Artifactualist approach, the epistemic value of models lies in their capacity to canalize certain courses of action through which to maintain the coherence of experience.

Second, this framework challenges the “model-target” dyad as the fundamental unit through which to understand and make sense of the epistemic role of models. Instead, the primary epistemic unit of analysis is the experience. Targetism is set aside since models are not already oriented to represent predefined worldly systems. Rather, the epistemic role consists in structuring activities through which problematic situations are transformed into determinate ones, reestablishing the coherence of experience.

To understand how this Pragmatist-Artifactualist framework restores framing inquiries granting a role to experimental model organisms in the exploration of new avenues of thought—particularly concerning the idea of inheritance—it is necessary to delve deeper into the two components underlying this proposal: the idea of experience and the notion of artifact.

Let us analyze the idea of experience. A defining feature of John Dewey’s philosophy is the rejection of dualism: there are no two self-contained entities, subjects and objects (Dewey & Bentley, 1946). The targets scientists seek to understand arise and become meaningful within how they experience the world; there is no knowledge outside it (Dewey, 2023a, p. 248). Importantly, experience should not be interpreted from a subjective or psychological point of view (Dewey, 1917). Rather, it refers to the dynamic and continuous process of transactions that takes place between agents and their environment: “experience as an interactivity of organic and environmental factors, is a rhythm of doings-undergoings which presents a serial alternation of relative upsets and recoveries, states of imbalance and reintegrations, unsettling and settlements” (Dewey, 2023a, p. 228). Influenced by biological research, Dewey interpreted experience through the idea of *vital function*—relations taking place between the organic agent and its environment (Dewey, 2023b, p. 315). For Dewey, these relationships are conceived through the idea of *transaction*, which differs from *interaction* in rejecting the existence of two self-subsistent entities that enter a relationship via opposition (Dewey & Bentley, 1946). Contrary to asymmetric externalism, transaction emphasizes the co-dependent character of the knower and the known—something stressed by organicists, see, e.g., Haldane, 1884—as well as the dynamism of this relationship.

Typically, the vital functions structuring a course of experience are in equilibrium: agents draw on a body of organized and well-established habits^11^ to functionally cope with environmental demands (MW14). Yet imbalances inevitably arise as living beings confront novel or shifting environmental conditions, calling into question the stability of the very body of organized habits—questions arise that cannot be solved by means of possessed habits. In scientific contexts, disruptions that threaten the stability of the practical course of experience are often termed problematic situations. It is critical to understand that these situations are problematic in and through the transactions that take place within a course of experience (LW4). We can only become aware of environmental stimuli that threaten the integrity of our practice by virtue of previous responses, of which the former are the consequence, and because of the need to employ the latter as a means of further action (LW1, p. 253). Recognizing a problematic situation generates research goals, understood by Dewey as stimuli for action: anticipated outcomes that guide inquiry with the aim of coherently reorganizing the cognitive environment (MW14).

Consider research on external intergenerational epigenetic inheritance. Within the organismal tradition of epigenetics, several situations have been considered problematic against the background of a core of theoretical assumptions, advancing goals to reestablish the coherence of the course of experience. Again, these questions only arise as problematic within this particular course of experience, being irrelevant to related research agendas such as the molecular epigenetic one focused on gene regulatory elements, germline/material overlap, and transgenerational stability. Consider some problematic questions regarding inheritance that may arise within the course of experience of organismal epigenetics:

1. What developmental processes regulate the (re)occurrence of particular adaptive traits?
2. When are these mechanisms at work? We know that external intergenerational epigenetic inheritance will not be the same for all organisms, traits, and ecological situations. In fact, it is to be expected that the scope of this form of inheritance will be narrow.
3. What are the time windows in which epigenetic processes can have a phenotypic impact? It is to be expected that the early stages of development, due to their high sensitivity and plasticity, are more sensitive to high-impact epigenetic modifications.
4. Does the environmental cue that triggers the epigenetic state require temporal stability? If so, for how long?
5. What level of specificity should exist between environmental triggers and induced epigenetic states, and how does this relate to the existence of generalist or specialized developmental plans?
6. How might epigenetic inheritance modulate evolutionary forces in the speciation process? It is possible that it may slow down evolution by selection due to the Boogert effect. It may also mitigate the importance of drift by reducing the possibility of bottlenecking (Greenspoon et al., 2022).

The goal of inquiry is to resolve the tensions by transforming these problematic situations into determinate ones, thereby restoring the unity and coherence of experience—i.e., the vital function incarnated in the functional relationship between “organism and environment” (MW14, LW4). This restoration occurs when the information generated through research enables the reorganization of the functional relations among prior habits, leading to coherent and effective actions within the research agenda (LW1; LW12, p. 520). On this view, scientific research is not primarily directed at establishing representational relations but at rendering problematic situations determinate, reestablishing experiential coherence where it has been disrupted. Epistemic artifacts are central to this process.

Artifactualism, as developed by Knuuttila and colleagues, holds that models are erotetic devices—artificial systems intentionally constructed by cognitive agents on the basis of existing knowledge to address specific scientific questions motivated by theoretical or empirical considerations (Knuuttila, 2005). In characterizing models as artifacts, Knuuttila emphasizes two key points of interest for present purposes.

First, she challenges a central representationalist assumption: that models are initially disconnected from their targets and must be subsequently linked through a representational relation. For Knuuttila, models are from the outset connected to the world because their construction is motivated by concrete scientific problems (Knuuttila, 2011, p. 267). Accordingly, understanding how models generate knowledge does not require analyzing the nature of the model–target relation; rather, it requires examining the modeling process itself—how intentionally built devices enable or constrain epistemic access to the phenomenon under investigation.

Second, and in connection with the previous point, she foregrounds the material dimension of models. Models are “intentionally constructed things that are materialized in some medium” (Knuuttila, 2005, p. 1266). In other words, they have a material dimension that shapes and restricts specific forms of inference (Knuuttila, 2017, p. 12). Knuuttila distinguishes between means (the material embodiment of the model) and modes (devices used to express the meaning of models) of presentation. Scientists select means and modes that best suit the questions, structuring their inquiry (Knuuttila, 2011, p. 263).

Considered from the suggested pragmatist standpoint,^12^ the epistemic function of artifacts does not lie in revealing objective features of pre-given targets subsisting *de re* (i.e., targetism) but in canalizing activities oriented to reestablish the coherence of habits and, hence, the functionality of the organism-environment relationship. In short, the Pragmatist-Artifactualist approach conceives models as artifacts employed to stabilize the course of experience.

*Def. Artifact.* It is a stable configuration, whether material or theoretical, whose components, properties, and relationships have been intentionally selected for the explicit purpose of rendering determinate an aspect that is considered problematic within a course of experience.

To gain the status of “artifacts”, a material or theoretical configuration must be inserted within a course of experience. Importantly, the agent does not need to create the artifact from scratch; pre-existing configurations can be co-opted. However, for the artifact to be recognized as relevant to satisfying a cognitive goal, it must be interpreted in light of the ongoing course of experience. Only then can the agent discern the advantages it offers in generating information that restores vital functionality. Crucially, the information obtained will depend on the material or structural characteristics that characterize the epistemic artifact, which will be, in turn, subordinated to the problematic situation, and hence the cognitive goal, to be answered.

Consider these analyses in the context of external intergenerational epigenetic inheritance. First, it is necessary to select organisms that display the material features conducive to eliciting the particularities of this mechanism. For example, clonal/asexual organisms or plants. For the former, environmentally induced epigenetic variation can facilitate the emergence of adaptive phenotypes that overcome the constraints imposed by low genetic diversity (Thorson et al., 2017). For the latter, their modular and indeterminate growth, the absence of an early segregated germline, and their extensive use of methylation and small RNA-mediated silencing make them prone to epigenetic-mediated plastic responses shaping their developmental trajectories. Importantly, a certain degree of sensitivity to the control of plastic processes must be exhibited by the organisms employed. As previously discussed, this is the case for asexual organisms and plants. However, similar considerations can be applied to other taxa. For example, environmental signals play a fundamental role in the phenotypic development of zebrafish (Norouzitallab et al., 2019; see also Moczek, 2010; Schwab et al., 2017). Second, attention needs to be paid to the ecological circumstances that organisms face. External intergenerational epigenetic inheritance may be relevant for organisms colonizing new environments (Vogt, 2023). Colonizing groups often consist of only a few specimens. Therefore, genetic diversity is low in the initial period of invasions, even if they are sexual organisms. Environmental adaptation through epigenetic mechanisms may provide a possible strategy to deal with this problem (Cavalieri, 2020; Vernaz et al., 2022). But not only that, it could also be relevant for organisms that inhabit changing environments. In particular, for sessile organisms, epigenetically mediated plastic alteration may enable the rapid generation of adaptive traits that allow them to cope with sudden environmental changes (Sultan, 2015). This generates a heuristic within the course of experience on how the experimental setup should be configured: to study this inheritance mechanism, it is essential to either recreate specific environmental conditions in the laboratory or conduct a field study. Finally, attention needs to be paid to the evolutionary history of organisms. Studying organisms that have moved rapidly from one ecosystem to another can be crucial to assessing the impact of this inheritance mechanism.

Apart from having the relevant material characteristics, the agent must have the necessary performative knowledge to manipulate the artifact and extract the information to restore the coherence of experience. The artifact does not yield significant information by itself. An experimental configuration only acquires the status of an artifact when users know how to employ it to demonstrate whether the initially handled hypothesis that would allow satisfying the problematic situation is adequate. In the case under consideration, one must have knowledge of techniques related not only to major epigenetic modifications (e.g., DNA analysis through bisulfite sequencing, digestion-based assays, chromatin immunoprecipitation (ChIP), or sequential ChIP-bisulfite-sequencing) but also to developmental processes (techniques for embryo manipulation and culture, cell lineage tracing, in vivo and ex vivo imaging of tissue and organ development, gene knockdown or knockout approaches, and methods for temporally and spatially controlled environmental perturbations).

In short, an experimental setup only becomes an artifact when the agent (a) recognizes the opportunities offered by the model and (b) possesses the knowledge to manipulate it effectively. If these conditions are not met, an experimental setup fails to acquire the status of an artifact and lacks epistemic value. This framework explains why different agents select different types of artifacts: the choice depends on the structure of the course of experience, the particular cognitive goals being pursued, and the performative skills.

It is crucial to recognize that scientists do not work with fully defined artifacts. Such a situation would presuppose prior knowledge of the phenomenon being addressed—i.e., the problematic situation—, reverting to targetism and collapsing in framed inquiry. Instead, researchers engage with provisional and perfectible devices designed to elicit answers not yet known to questions that are not yet fully articulated (Rheinberger, 2023, p. 104). The epistemic value of artifacts does not reside in their capacity to represent a phenomenon but in their ability to scaffold a set of activities that yield information capable of restoring the coherence of experience. There is a dialectical relationship between hypotheses and artifacts: hypotheses guide the initial construction of artifacts, while the manipulation of these artifacts, in turn, renders the hypotheses intelligible, stabilizing features of the problematic situation and progressively refining both the artifact and the conceptual understanding. This iterative process continues until the necessary information is obtained to re-establish the coherence of the habits and the functionality of the organism-environment relationship, leading to coherent and effective actions within the research agenda.

From this perspective, representation is reconceived. To represent a phenomenon is not to capture a pre-defined target but to re-present an aspect of the experienced world perceived as problematic that is initially ill-defined under a particular experimental configuration, to specify it and eventually make it intelligible. The fundamental goal is to transform situations deemed problematic into more determinate ones. This transformation involves the construction/co-optation and use of artifacts. This approach overcomes a central limitation of representationalism: its inability to promote the practical investigation of loosely delineated hypotheses/models that have the potential to advance our understanding of reality.

Before concluding, let me recapitulate how this Pragmatist-Artifactualist framework provides space for framing inquiry, enabling experimental model organisms to realize their epistemic potential in testing a disruptive hypothesis, such as external intergenerational epigenetic inheritance. Due to its extremely unique characteristics, assessing the scope and impact of this mechanism requires very specific methodological designs that align with the presumed particularities (i.e., externality and intergenerationality) and with certain background knowledge associated with an organismal agenda (theoretical and experimental). Setting aside targetism, the ability of an organism to faithfully represent the putative target system should not be the main parameter in choosing an organism for research. The key element will consist in the selection of the organism that displays the material, ecological, or evolutionary features to obtain the type of information that will allow us to render intelligible the problematic situation within the very course of experience: namely, the presumed existence of a mechanism of inheritance that calls into question the widely accepted conditions of molecularism, germline/material overlap and transgenerational stability. In this case, we know that adaptive responses may be more effective when drawing on plastic processes. We also know that this route to adaptation may be particularly important when genetic variation is lacking, such as in small, bottlenecked, or asexual populations or in organisms facing particular environmental—e.g., the sudden environmental changes—or evolutionary scenarios—e.g., a rapid transition from one niche to another. With these variables in mind, it is necessary to select those organisms that instantiate them. The case studies cited in Sect. 2. provide an excellent illustration.

## Conclusion

In this paper, I have argued that a central step in exploring innovative avenues of thinking involves shifting the research focus from model organisms to experimental model organisms. To defend this point, I have shown how experimental model organisms may allow us to investigate a singular epigenetic mechanism that challenges major assumptions about inheritance: external intergenerational epigenetic inheritance. I have argued that reorienting our practices towards experimental model organisms is insufficient since the representational role traditionally attributed to experimental model organisms still poses a serious epistemic obstacle. Considering the case study mentioned above, I have illustrated how the misgivings surrounding external intergenerational epigenetic inheritance stem not from its theoretical implausibility but from the lack of experimental evidence—evidence that has failed to materialize because of representationalist biases. To overcome this problem, I have outlined an alternative Pragmatist-Artifactualist approach to restoring framing inquiry for experimental model organisms. From pragmatism, I took the idea of experience as the fundamental unit of analysis in understanding the epistemic status of models. From artifactualism, I borrowed the idea that the epistemic role of models consists in structuring particular activities through which problematic situations are transformed into determinate ones, reestablishing the coherence of the course of experience.

## Footnotes

1 This alignment does not preclude debates over specific results, interpretations, or theoretical approaches. I thank the reviewer for prompting me to clarify this point.

2 It is important not to conflate an experimental model organism with a non-model organism. Although non-model organisms lack the degree of standardization and institutionalization characteristic of model organisms, they still play the epistemic role of supporting generalization (enlarging comparative diversity). In contrast, an experimental model organism is selected because its exceptional properties make it optimal for addressing a specific problem, without any initial intention to generalize the results.

3 Determining which organism is most appropriate for investigating a particular conserved process is fundamentally a context-dependent question. The purpose of this article is not to adjudicate such choices, but rather to clarify the distinct epistemic and pragmatic dimensions that characterize the use of model organisms versus experimental model organisms.

4 It must conform to widely accepted background assumptions.

5 It must be an accepted and standardized organism with a record of success in addressing the question at stake.

6 The use must be adjusted to particular funding strategies.

7 There should be a specialized and well-established infostructure to share information.

8 The term “epigenetic” is used here in the broad historical sense derived from the theory of epigenesis, which conceives organismal form as emerging through processes of differentiation and integration operating across multiple levels of regulation and in continuous interaction with environmental conditions (Peterson, 2016). More specifically, it refers to the way this perspective was articulated within several strands of late nineteenth- and early twentieth-century organicist thought. Accordingly, the term is not employed in the later, more restricted sense associated with C. H. Waddington, who incorporated epigenetic ideas into a more gene-centered framework of development (Loison, 2022). I thank the reviewer for prompting me to clarify this point.

9 Adherence to the representational function transcends these two theoretically inflated variants—associated with additional ontological or epistemic commitments—reaching deflationary approaches (Frigg & Nguyen, 2020; Suárez, 2024, p. 78).

10 This point is particularly salient in the case of Sanches de Oliveira’s view, which is grounded in the same intellectual tradition I draw on here—Deweyan philosophy and artifactualism.

11 Stable patterns of perception and action built around theoretical and performative knowledge grounded in past experiences that govern our thoughts and shape our actions (MW14).

12 Interpreted from this pragmatist standpoint, the artifactual approach dispenses with all representational commitments—see Sanches de Oliveira (2022) and Sánchez-Dorado (2024) on the residual representationalism present in standard artifactualism.
